# Dual Flame-Retardant and Curing-Agent Effects of Phytic Acid–Guanazole as an Additive in Fire-Protective Coatings for Wood

**DOI:** 10.3390/polym17091169

**Published:** 2025-04-25

**Authors:** Xue Zheng, Yongjin Zou, Cuili Xiang, An Wei, Yuhong Wei, Lixian Sun

**Affiliations:** 1 School of Materials Science and Engineering, Guilin University of Electronic Science and Technology, Guilin 541004, China; 2 Guangxi Zhiguang Office Furniture Co., Ltd., Liuzhou 545006, China

**Keywords:** bio-based flame retardant, urea–formaldehyde resin, phytic acid, guanazole

## Abstract

Recent research has focused on developing environmentally friendly flame-retardant coatings to improve the fire resistance of wood. In this study, phytic acid–guanazole (PG), a dual-functional compound synthesized through an ionic reaction between phytic acid and guanazole, was added to KH550-modified urea–formaldehyde resin (KUF) as both a curing agent and flame retardant. The $-{\mathrm{PO}}_{4}^{3-}$ groups from phytic acid act as an acid source to accelerate char formation during combustion, while the −NH_2_ groups introduced by guanazole release non-combustible gases to dilute oxygen in the air, synergistically enhancing flame retardancy. Additionally, the hygroscopic $-{\mathrm{PO}}_{4}^{3-}$ groups absorb free water in the resin, reducing the curing temperature and accelerating coating solidification. The KH550 coupling agent improves compatibility between KUF and PG while introducing silicon, which forms SiO_2_ during combustion to strengthen the char layer and further enhance flame resistance. Evaluations showed that PG outperforms conventional tannic acid (TA) in curing efficiency and fire resistance. Comprehensive analyses, including Differential Scanning Calorimetry (DSC), Limiting Oxygen Index (LOI), vertical flame tests, and cone calorimetry, confirmed PG’s dual functionality. Scanning Electron Microscope (SEM) and Raman spectroscopy revealed that PG-modified coatings form denser post-combustion char layers, directly linked to improved fire resistance. As a multifunctional additive, PG eliminates the need for separate curing agents and utilizes bio-based phytic acid, offering cost-effective and sustainable advantages for industrial applications.

## 1. Introduction

Wood is a commonly used resource that has extensive applications. However, it is necessary to enhance the flame retardancy of wood. To improve the flame resistance of wood, it is commonly impregnated with a flame-retardant chemical. In a recent study, Fan et al. [1] incorporated ammonium dihydrogen phosphate (ADP) into wood cell structures through an impregnation-based approach, as detailed in their research. The decomposition of ADP accelerates the formation of insulating char, which restricts heat transfer from the wood surface to its interior. The densely cross-linked wood structure leads to the formation of a condensed char structure that limits the escape of pyrolytic flammable gases. This combination of insulation and condensed char effectively protects the internal wood structure and maintains its mechanical properties against high-temperature flame heating. The developed char layer acts as a thermal and oxygen barrier to achieve flame retardation. Applying a surface coating of a flame-retardant paint to wood is another common approach. Chen et al. developed an epoxy-modified silicone resin (SiR) coating through the condensation of dimethyldiethoxysilane with 3-glycidyloxypropyltrimethoxysilane (KH-560) [2]. The SiR resin was cured using PTDP, a nitrogen–phosphorus flame retardant (synthesized from polyethyleneimine, thiourea, and diphenylphosphinic chloride) serving as an intrinsic curing agent. This system enhances flame retardancy by generating silica during combustion, which improves the integrity of the char layer, acting as a barrier to oxygen and heat transfer, thereby significantly improving the fire resistance of wood. Although this coating significantly improved flame retardancy, the PTDP curing agent exhibited poor environmental degradation, posing potential pollution risks during long-term use. Whether ADP is introduced into wood through impregnation or PTDP is incorporated into flame-retardant coatings applied to wood, the fundamental mechanism relies on the introduction of nitrogen (N) and phosphorus (P) elements. N acts as a gas source, generating non-combustible gases during combustion to dilute oxygen concentration, while P serves as an acid source, accelerating and enhancing the formation of a high-quality char layer. Their synergistic interaction significantly improves the material’s flame-retardant performance. Tannic acid, a non-toxic, cost-effective, and readily available polyphenolic compound [3], exhibits acidity due to hydroxyl groups bonded to its aromatic rings. Its unique aromatic structure imparts high thermal stability, while its inherent carbon content acts as a carbon source during combustion to enhance char layer formation. Simultaneously, the hydroxyl groups serve as an acid source to improve char quality and enable its dual functionality as a curing agent in coatings, making it a widely adopted curing agent in flame-retardant coatings.

Urea–formaldehyde (UF) resin, a thermosetting polymer, is extensively utilized as an adhesive or coating in wood product manufacturing due to its low-cost synthesis, high hardness, exceptional water resistance, and robust adhesion properties [4,5,6]. However, due to the inherent flammability and fire hazard of UF resin, enhancing its flame retardancy to improve the fire resistance of UF resin-coated wood products has emerged as a critical research focus in materials science [7,8].

In recent years, bio-based flame retardants have garnered significant attention as sustainable alternatives to traditional flame retardants, due to their low cost, renewability, and biodegradability [9]. Phytic acid, a bio-based compound with high phosphorus content and strong acidity, is one of the most promising raw materials for bio-based flame retardants. Its high phosphorus content enhances the quality of the char layer, while its strong acidity promotes char layer formation in materials. However, the high acidity of phytic acid (with a pH value of approximately 1.2) can degrade the mechanical properties of flame-retardant coatings, rendering them brittle and prone to cracking and detachment. To mitigate this issue, alkaline materials can be used to neutralize phytic acid, thereby reducing its acidity and improving its mechanical properties [10,11]. Guanazole, a highly nitrogenous (58.3 wt.% nitrogen) hygroscopic monobasic alkali, can effectively neutralize the acidity of phytic acid. Moreover, guanazole contains ammonium ions, which release non-combustible gases (such as NH₃) during thermal decomposition. These gases dilute the oxygen concentration and enhance the flame-retardant performance of the material [12].

In this study, a phytic acid–guanazole (PG) urea–formaldehyde resin coating with dual flame-retardant and curing functions was synthesized. Phytic acid was selected as the acid source to introduce a large number of phosphate ions, which promote char layer formation and enhance its quality. Guanazole neutralizes the acidity of phytic acid, thereby reducing brittleness and suppressing cracking and delamination. Additionally, it introduces ammonium ions to increase the nitrogen content in the flame retardant system. The nitrogen acts as a gas source to generate non-combustible gases during combustion, diluting the oxygen concentration in the air. These two actions work synergistically to enhance the flame-retardant performance of the material.

The selection of PG as a flame-retardant curing agent offers distinct advantages over conventional counterparts. Compared to polyphosphates, phytic acid provides dual advantages: (1) its plant-derived origin, renewability, and biodegradability significantly reduce environmental impact, aligning with green chemistry principles; (2) the six phosphate groups in its molecular structure provide exceptional chelation capacity for efficient binding with ammonium ions. Compared to melamine—a common raw material in traditional ammonium phosphate-based flame retardants—guanazole exhibits superior water solubility, enabling more efficient reaction processes. Furthermore, its thermal decomposition releases inert gases (e.g., nitrogen, ammonia), achieving high flame-retardant efficiency with minimal residue, while demonstrating enhanced biodegradability, safety, and compliance with sustainable chemistry.

During the preparation of the urea–formaldehyde resin, KH550 (γ-aminopropyltriethoxysilane) was introduced as a coupling agent. This modification enhances the compatibility between the resin and the flame-retardant curing agent PG while incorporating silicon into the system. During subsequent combustion, the silicon forms oxides such as silicon dioxide, which further increases the density of the char layer, thereby enhancing the coating’s flame-retardant performance.

Moreover, PG can also serve as a curing agent for the coating. It achieves the same curing effect as conventional curing agents without the need for additional additives, thereby reducing costs while maintaining flame-retardant performance.

## 2. Materials and Methods

### 2.1. Materials

Poplar solid wood board was purchased from Linshi Wood Industry Direct Sales Store, China. Phytic acid (PA; 70 wt.% aqueous solution, CAS No. 83-86-3), formaldehyde solution (37 wt.% in H₂O, CAS No. 50-00-0), urea (CAS No. 57-13-6), dioctyl phthalate (CAS No. 117-81-7), guanazole (GZ; CAS No. 1455-77-2), γ-aminopropyltriethoxysilane (KH550, CAS No. 919-30-2), and tannic acid (TA; CAS No. 1401-55-4) were sourced from Aladdin Scientific Corp. (Shanghai, China). Deionized water (18.2 MΩ·cm) was used to prepare various solutions, including aqueous solutions of sodium hydroxide (NaOH, 20 wt.%) and acetic acid (20 wt.%).

### 2.2. Sample Preparation

#### 2.2.1. Synthesis of PG

Under ambient conditions, 13.5 g of GZ was dissolved in 100 mL of deionized water to prepare a 1.204 mol/L guanazole aqueous solution. In addition, 15 mL of a 70 wt.% PA aqueous solution was diluted in 50 mL of deionized water to obtain a 0.325 mol/L PA aqueous solution. The PA solution was then added dropwise to the GZ solution at a controlled rate of 0.5 drop/s under continuous mechanical stirring (500–600 rpm) to ensure a complete reaction between GZ and PA. The resultant mixture was subjected to solvent removal using a rotary evaporator (40 °C, 0.09 MPa) to yield solid PA–GZ. The obtained product was pulverized into a homogeneous powder using an agate mortar and is henceforth denoted as PG.

#### 2.2.2. Synthesis of KH550-Modified UF Resin

First, 37 wt.% formaldehyde solution (100 g) was precisely weighed, adjusted to pH 8.0–8.5 using 20 wt.% NaOH aqueous solution, and transferred into a three-necked flask equipped with a mechanical stirrer. The mixture was heated in a water bath under continuous stirring at 400 rpm until the temperature stabilized at 90 °C. At this temperature, the first batch of urea (37 g) and KH550 (2.85 g) was sequentially added. The reaction was maintained under stirring for 30 min. Subsequently, the pH was carefully adjusted to 4.5–5.0 with 20 wt.% acetic acid aqueous solution, followed by the addition of the second batch of urea (12.3 g). Stirring continued for 10 min until turbidity developed throughout the solution. The pH was then neutralized to 7.5–8.0 using 20 wt.% sodium hydroxide aqueous solution, and the third batch of urea (7.6 g) was introduced. The temperature was lowered to 70 °C, and the mixture was stirred for an additional 30 min to ensure complete polycondensation. Finally, the synthesized UF resin solution was collected and cooled to ambient temperature. The UF resin modified with KH550 is named KUF.

#### 2.2.3. Fabrication of Flame-Retardant Wood Samples

The flame-retardant coating solution was prepared by mixing 100 g of KUF resin solution with 1 g of dioctyl phthalate and varying amounts of PG (1.0, 1.5, 2.0, or 2.5 g) as a curing agent, followed by stirring at 500–600 rpm for 30 min. The pH was then adjusted to 4.0–5.0 by alternately adding 20 wt.% acetic acid and 20 wt.% NaOH aqueous solutions to obtain the KUF resin-based flame-retardant coating. The experimental groups were designated as KUF-PG-x, where x indicates the amount of curing agent added (1–2.5 wt.%). For example, the KUF-PG-1 sample contains 1 wt.% PG as the curing agent. For comparison, a control solution (KUF-TA-2.5) with identical composition to the experimental groups (similarly pH-adjusted to 4.0–5.0) was prepared to produce the KUF resin flame-retardant coating. The formulated coatings were subsequently applied to wood substrates pre-cut into specified dimensions, achieving a coating thickness of approximately 0.3 mm. The coated wood samples were cured under ambient conditions for 24 h to obtain flame-retardant wood specimens.

### 2.3. Sample Characterization

#### 2.3.1. Structural Characterization and Thermal Analysis

The cured fire-retardant coating material was ground into a powdered form, and the resulting powder sample was subsequently subjected to further testing. Fourier transform infrared (FTIR) spectroscopy was performed using a TENSOR27 spectrometer (Bruker, Saarbrücken, Germany). The microstructure and elemental distribution of the residual carbon layer were examined by scanning electron microscopy (SEM) using a Quanta 450 field-emission system (FEI, Hillsboro, OR, USA) coupled with energy-dispersive spectroscopy (EDS). Raman spectra were obtained over a wavenumber range of 400–2000 cm⁻^1^ using a LabRAM HR Evolution Raman spectrometer (Horiba Scientific, Paris, France) equipped with a 532 nm laser. The curing behaviors of the fire-retardant coating materials were analyzed by DSC using an STA 449 F3 thermal analyzer (Netzsch, Saarbrücken, Germany) under a nitrogen atmosphere. The samples were tested over a temperature range of 40–180 °C with a heating rate of 10 °C/min. Thermogravimetry Analysis (TGA) was performed using an IS 50 thermogravimetric analyzer (Thermo Fisher Scientific, Waltham, MA, USA) over a temperature range of 50–800 °C with a heating rate of 20 °C/min under a nitrogen flow rate of 100 mL/min.

#### 2.3.2. Flammability Tests

The wood pieces were cut into three geometries: 125 mm × 12.5 mm, 125 mm × 6.5 mm, and 100 mm × 100 mm, with a nominal thickness of 3 mm. A fire-retardant coating layer (~0.3 mm thick) was uniformly applied to the surfaces. After ambient-temperature curing (The processed wood is uniformly named W-KUF-XX-X in the following text), the specimens were evaluated through vertical flammability tests, limiting oxygen index (LOI) measurements, and cone calorimetry. Vertical flammability tests were conducted using a CZF-3 vertical flame tester (Jiangning Analysis Instrument Co., Ltd., Nanjing, China); LOI measurements were performed with an HC-2 oxygen index meter (Jiangning Analysis Instrument Co., Ltd., Nanjing, China); and cone calorimetry tests were carried out using a TTech-GBT161172 cone calorimeter (Testech Technology, Suzhou, China).

#### 2.3.3. Post-Combustion Char Assessment

The char residue layer collected after cone calorimeter combustion testing was subjected to further analysis. The microstructure and elemental distribution of the residual carbon layer were examined by scanning electron microscopy (SEM) using a Quanta 450 field-emission system (FEI, Hillsboro, OR, USA) coupled with energy-dispersive spectroscopy (EDS). Raman spectra were obtained over a wavenumber range of 400–2000 cm⁻^1^ using a LabRAM HR Evolution Raman spectrometer (Horiba Scientific, Paris, France) equipped with a 532 nm laser.

## 3. Results and Discussion

### 3.1. Synthesis and Characterization for PG and Flame-Retardant Coating Samples with Different Formulations

**Figure 1:**
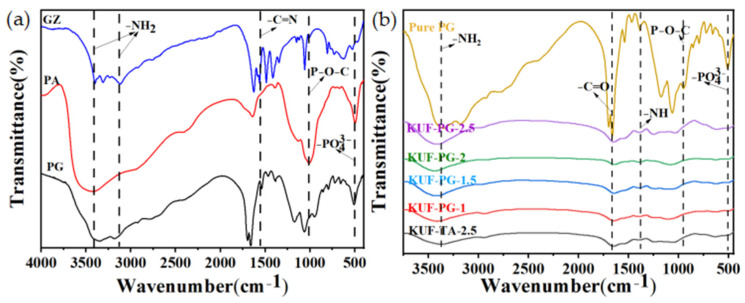
FTIR spectra of (**a**) GZ, PA, and PG, and (**b**) PG and flame-retardant coating samples with different formulations.

Figure 1a illustrates the FTIR spectrum of the PG flame-retardant curing agent and its constituent components (GZ and PA). Characteristic absorption bands are observed at 3408 cm^−1^ (N–H stretching vibrations of –NH_2_ groups) [13], 500 cm^−1^ (symmetric stretching of –${PO}_{4}^{3-}$) [14], and 1022 cm^−1^ (P–O–C stretching vibrations) [15], collectively confirming the successful synthesis of PG. The FTIR spectra of PG and the flame-retardant coating materials in Figure 1b illustrate distinct structural features. The FTIR spectrum of pure PG exhibits a C = O stretching band at 1656 cm^−1^ [16] and a P–O–C vibration band at 967 cm^−1^, while the spectra of all coating samples retain −NH_2_, −NH, and C = O vibrations originating from the KH550-modified UF resin matrix. Notably, the KUF-PG-2.5 spectrum demonstrates significantly enhanced absorption intensities at 1022 cm^−1^ (P–O–C) and 500 cm^−1^ ($-{\mathrm{PO}}_{4}^{3-}$) compared to other formulations, unequivocally verifying the effective incorporation of P-containing functional groups into the coating system.

### 3.2. Thermal Performance of PGKUFs

**Figure 2:**
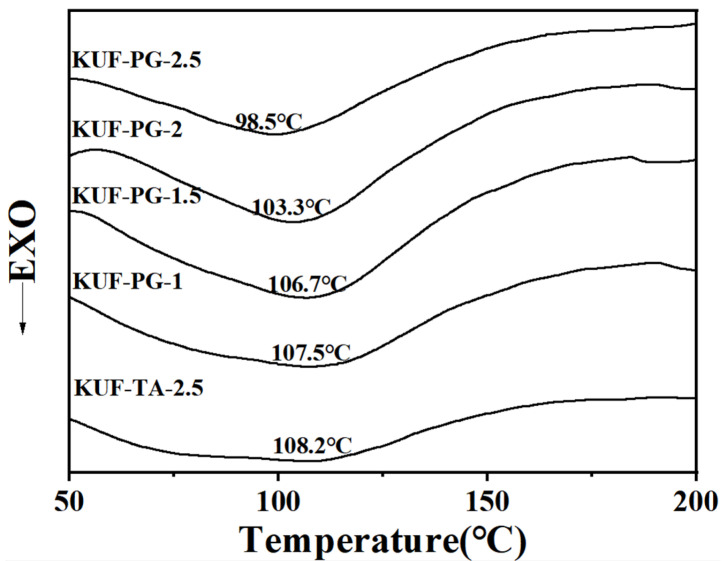
DSC curves of flame-retardant coating samples with different formulations.

Figure 2 displays the DSC results of the flame-retardant coating samples. All formulations exhibited the same curing trend in their curves, with only a single curing peak. Notably, the curing temperature decreased as the concentration of the PG curing agent increased. Furthermore, coatings using PG as the curing agent consistently demonstrated lower curing temperatures compared to those containing TA as the conventional curing agent, indicating that PG effectively reduces the curing temperature of KUF resin. The primary reason is attributed to the strong hygroscopicity of the phosphate groups in phytic acid, which absorbs free water in the resin system, thereby lowering the curing temperature of KUF.

**Figure 3:**
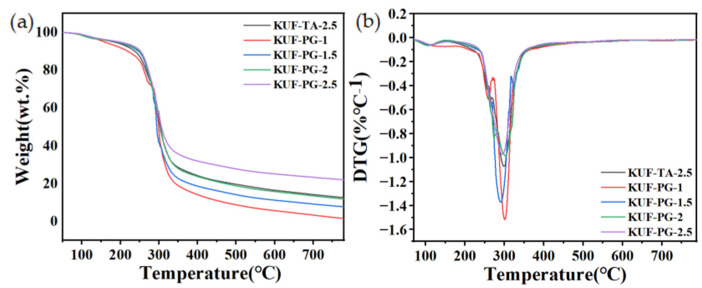
(**a**) TGA and (**b**) DTG curves of different flame-retardant coatings.

TGA/DTG tests were conducted to investigate the thermal decomposition behavior of the various flame-retardant coatings and the results are illustrated in Figure 3. These curves demonstrate that as the PG content increases, the final residual carbon content also significantly increases, so the thermal stability of the coatings improves with increasing PG content. To further analyze the thermal decomposition characteristics, the key data extracted from the TGA tests are summarized in Table 1. Increasing the PG content significantly enhances the temperature at 5% mass loss (*T_d_*_5%_, °C) and the residual char yield at 800 °C (*R*_800_, wt.%). Under identical curing-agent contents, the PG-based coatings exhibit higher *T_d_*_5%_ and *R*_800_ values than the TA-based coatings. These results confirm that replacing TA with PG improves the thermal stability, with the maximum enhancement observed at the maximum PG content. However, the temperature of the maximum mass loss rate (*T_max_*, °C), that is, the main peak in the DTG curves, is similar for all samples, indicating that the underlying decomposition mechanism is preserved despite the improved stability.

### 3.3. Flame-Retardant Performance of PGKUFWs

#### 3.3.1. Heat Release Rate and Total Heat Release

**Figure 4:**
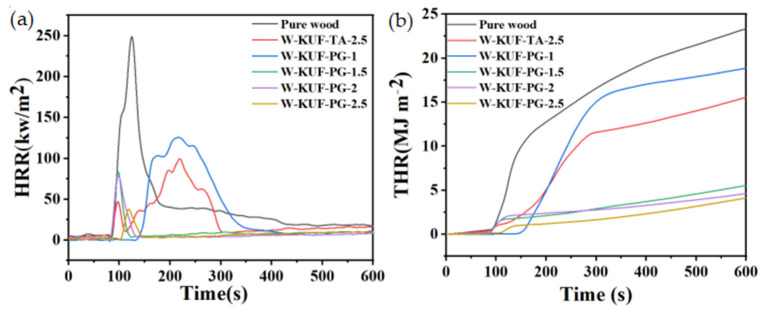
(**a**) HRR and (**b**) THR curves of various samples vs. time.

Figure 4 illustrates the cone calorimeter combustion test results. Figure 4a illustrates the heat release rate (HRR) during combustion. The untreated pure wood exhibits the highest HRR of 250 kW/m^2^. Upon applying the flame-retardant coatings, the HRR decreases significantly and progressively with increasing PG content. When the PG content reaches 2.5 wt.%, the peak HRR drops to 38 kW/m^2^, corresponding to an 84.8% reduction compared to that of untreated wood. Figure 4b illustrates the total heat release (THR) curves, which align with the HRR trends. With increasing PG content, the THR decreases markedly. After 600 s of combustion, the wood coated with the 2.5 wt.% PG-modified flame-retardant coating reaches a final THR of only 4.1 MJ/m^2^, which is substantially lower than that of the other samples.

Furthermore, at equivalent curing-agent concentrations, the HRR and THR values of the PG-based coatings are significantly lower than those obtained using TA as the curing agent. This finding demonstrates that PG exhibits superior flame retardancy compared to the conventional curing agent TA.

#### 3.3.2. Vertical Flammability and LOI Results

**Figure 5:**
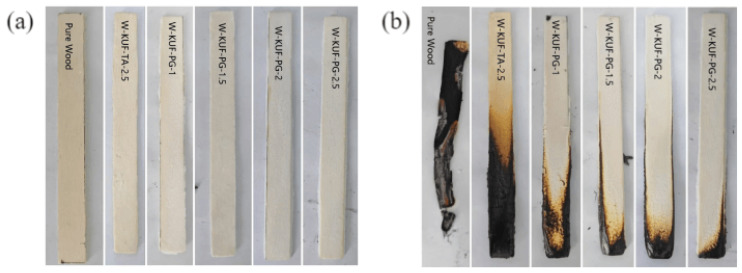
Photographs of samples (**a**) before and (**b**) after vertical flame tests.

Photographs of wood samples treated with the different flame retardant coatings before and after vertical flammability tests are illustrated in Figure 5. The treated wood samples exhibit significantly enhanced fire resistance compared to the untreated wood. To quantify the improvements in LOI and UL-94 ratings achieved by the different coating formulations, the corresponding test results are summarized in Table 2. The wood samples treated with the flame-retardant coatings exhibit significant improvements in both LOI and UL-94 ratings. However, the conventional TA-based coating achieved only a UL-94 V-1 rating, whereas the coatings formulated with PG as the curing agent demonstrated markedly enhanced performance. With increasing PG content, the UL-94 rating progresses from V-1 to V-0, accompanied by a simultaneous increase in the LOI values. Notably, at an equivalent curing-agent content (2.5 wt.%), the PG-based coatings outperform the TA-based system in terms of both LOI and UL-94 ratings. These findings show that replacing TA with PG as the curing agent significantly enhances the flame retardancy, confirming the cone calorimeter and thermogravimetric results.

### 3.4. Analysis of Char Residue After Cone Calorimetry Combustion

**Figure 6:**
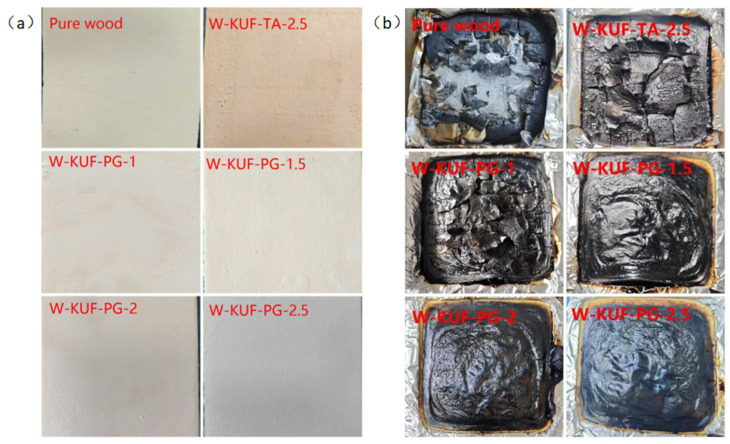
Photographs of wood samples (**a**) before and (**b**) after cone calorimetry combustion.

**Figure 7:**
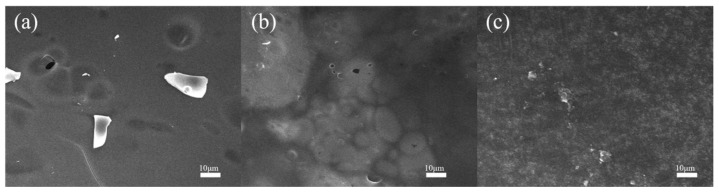
SEM images of the char layers formed after the combustion of (**a**) W-KUF-TA-2.5, (**b**) W-KUF-PG-1, and (**c**) W-KUF-PG-2.5.

Figure 6 illustrates photographs of wood samples before and after cone calorimetry combustion. The residual char layers observed in the photographs demonstrated progressively enhanced surface smoothness and integrity with increasing PG content, indicating improved char layer quality at higher PG content. To further investigate the char layer quality, the residual char layers of KUF-TA-2.5, KUF-PG-1, and KUF-PG-2.5 were characterized by SEM, as illustrated in Figure 7 (SEM). The residual char layer formed by the flame-retardant coating prepared using the traditional curing agent TA exhibits numerous pits and pores. In contrast, even when the content of PG is lower than that of TA (2.5 wt.%), the residual char layers of the PG-based flame-retardant coatings demonstrate superior quality, with no visible pits and significantly fewer pores. At the highest PG content of 2.5 wt.%, the highest-quality char layer is observed. The results of the comparative analysis demonstrate that replacing TA with PG significantly improves the quality of the residual char layer. The dense and continuous char layers formed by the combustion of the PG-based flame-retardant coatings effectively block oxygen and heat transfer, contributing to their superior flame-retardant performance.

**Figure 8:**
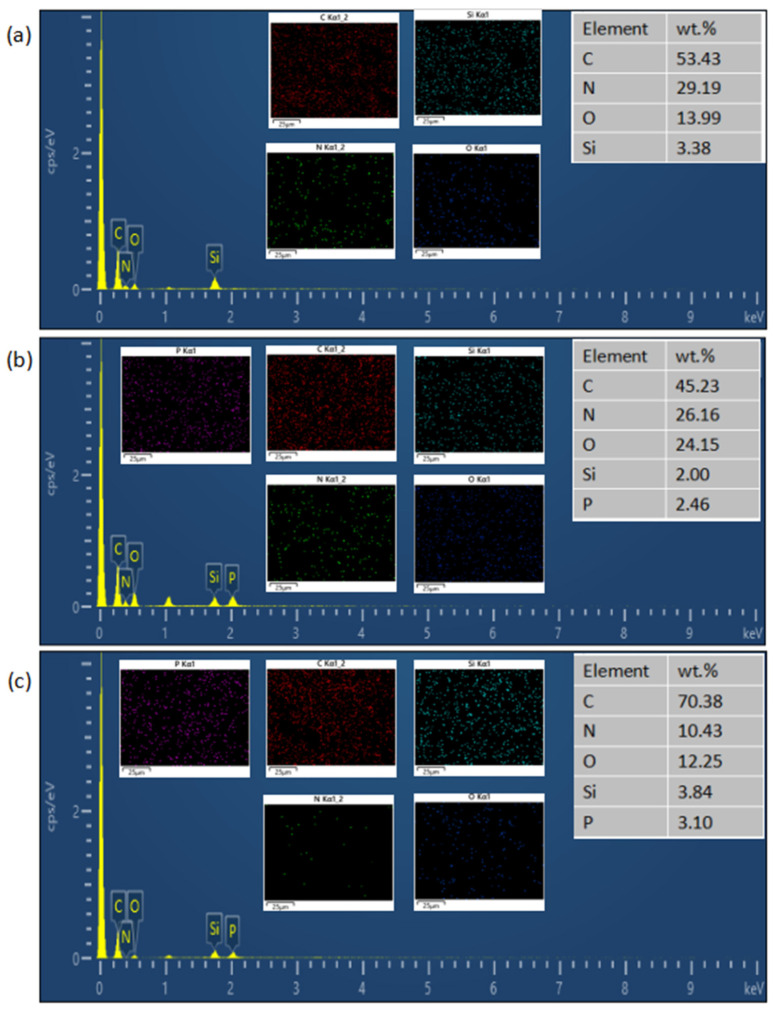
EDS images of the char layers formed after the combustion of (**a**) W-KUF-TA-2.5, (**b**) W-KUF-PG-1, and (**c**) W-KUF-PG-2.5.

To further analyze the surface elemental composition, EDS characterization was performed on the residual char layers of KUF-TA-2.5, KUF-PG-1, and KUF-PG-2.5 (Figure 8). Si was detected on all three samples, and the presence of SiO_2_ in the residues was explicitly confirmed, demonstrating its in situ formation during combustion. This provides direct evidence that the introduction of KH550 effectively promotes SiO_2_ generation in the post-combustion process, thereby improving the char layer quality (e.g., enhanced compactness and thermal stability).

**Figure 9:**
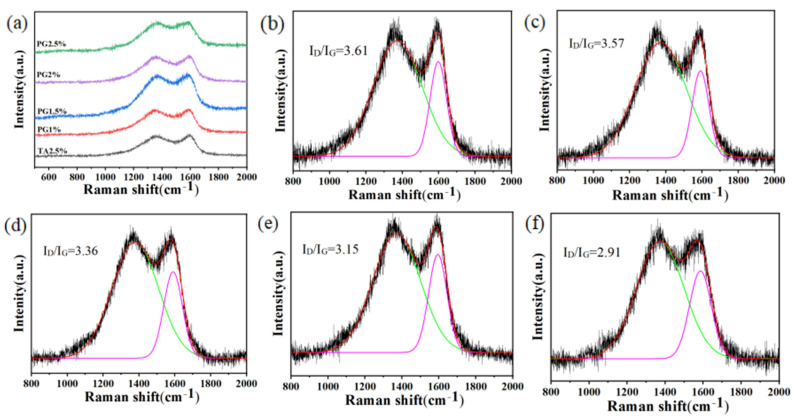
Raman spectra of the residual char layers after the combustion of various flame-retardant coatings: (**a**) comparison of wide-band spectra; spectra with fitted D and G bands for (**b**) W-KUF-TA-2.5, (**c**) W-KUF-PG-1, (**d**) W-PG-KUF-1.5, (**e**) W-KUF-PG-1.5, and (**f**) W-PG-KUF-2.5.

Raman spectroscopy was performed on the post-combustion char layers from the cone calorimeter tests to further investigate the chemical properties of the residual char (Figure 9). To characterize the quality of the carbon in the char layers, the characteristic D and G bands in the Raman spectra were compared. The peaks of the D and G bands are observed at 1372 cm⁻^1^ and 1595 cm^−1^, which represent disorder and defects in the graphitic structure and the ordered sp^2^ carbon structure, respectively. The intensity ratio of the D and G bands (*I*_D_/*I*_G_), or the *R*-value, is inversely proportional to the degree of graphitization. Based on the deconvolution of the Raman spectra, the calculated *R*-values are 3.61 for W-KUF-TA-2.5, 3.57 for W-KUF-PG-1, 3.36 for W-KUF-PG-1.5, 3.15 for W-KUF-PG-2, and 2.91 for W-KUF-PG-2.5. These results demonstrate that the residual char from coatings using PG as the curing agent exhibits a higher degree of graphitization compared to that formed from the traditional TA-based system. Furthermore, the *R*-value decreases with increasing PG content, indicating enhanced graphitization and char-layer quality. These findings support the conclusions drawn from previous tests, confirming the superior flame-retardant performance of PG-modified coatings.

## 4. Conclusions

In this study, a flame-retardant curing agent (PG) was synthesized through an ionic reaction between phytic acid and guanazole, and subsequently incorporated into KH-550-modified urea–formaldehyde resin (KUF) to prepare flame-retardant wood coatings. The synthesized PG exhibited dual functionality in the final resin system, serving as both a curing agent and a flame retardant. Notably, PG eliminated the need for conventional curing agents like TA while achieving superior curing performance. This enhancement is primarily attributed to the hygroscopic $-{\mathrm{PO}}_{4}^{3-}$ groups introduced by PG, which reduce the curing temperature of KUF and promote effective curing. Furthermore, the flame-retardant properties of PG were systematically investigated. Results demonstrated that the addition of PG increased the LOI of wood from 20.5% (untreated) to 32.7%, elevated the UL-94 rating from no classification to V-0, and reduced the peak heat release rate (HRR) and total heat release (THR) by 82.25% and 84.8%, respectively, compared to untreated wood. These improvements are ascribed to the synergistic effects of the ${PO}_{4}^{3-}$ and −NH_2_ groups introduced by PG. The $-{PO}_{4}^{3-}$ groups act as an acid source, facilitating dehydration and carbonization during combustion to form a thermally insulating char layer that blocks heat and oxygen. Meanwhile, the −NH_2_ groups release non-combustible gases, diluting oxygen concentration in the combustion zone. Additionally, KH-550 not only improved the compatibility between PG and KUF but also introduced silicon (Si), which forms SiO_2_ during combustion to reinforce the char layer and enhance flame retardancy. Remarkably, PG’s dual role as both a curing agent and flame retardant in the KUF system not only improved fire resistance but also reduced curing costs. Moreover, PG, derived from bio-based phytic acid and synthesized via a simple process, demonstrates significant potential for industrial applications due to its cost-effectiveness and environmental sustainability.

## Figures and Tables

**Table 1:** TGA and DTG data for different coatings.

Sample	*T_d_*_5%_ (°C)	*T_max_* (°C)	*R*_800_
KUF-TA-2.5	181.3	298.7	12.5
KUF-PG-1	157.6	300.3	1.3
KUF-PG-1.5	188.5	289.4	7.6
KUF-PG-2	190.1	302.7	11.7
KUF-PG-2.5	195.3	294.8	21.9

**Table 2:** UL-94 and LOI data of different woods.

Sample	LOI (%)	t_1_: After-Flame Time (1st Ignition) (s)	t_2_: Total After-Flame + Afterglow Time (2nd Ignition) (s)	Dripping Occurrence	Cotton Ignition	UL-94 Rating
Pure Wood	20.5	>60	-	Yes	Yes	No
KUF-TA-2.5	31.5	0.9	34.7	No	No	V-1
KUF-PG-1	28.8	2.2	24.5	No	No	V-1
KUF-PG-1.5	29.9	1.4	12.3	No	No	V-0
KUF-PG-2	32.1	1.2	3	No	No	V-0
KUF-PG-2.5	32.7	1.2	1.3	No	No	V-0

## Data Availability

The original data for this study are included in the article. For any further inquiries, please contact the corresponding author.
